# Dopamine receptor alterations in female rats with diet-induced decreased brain docosahexaenoic acid (DHA): interactions with reproductive status

**DOI:** 10.1179/147683010X12611460764282

**Published:** 2010-08

**Authors:** Paul F. Davis, Marlies K. Ozias, Susan E. Carlson, Gregory A. Reed, Michelle K. Winter, Kenneth E. McCarson, Beth Levant

**Affiliations:** 1 Department of Pharmacology, Toxicology, and Therapeutics; 2 Department of Dietetics & Nutrition; 3 Department of Pediatrics; 4 Kansas Intellectual and Developmental Disabilities Research Center, University of Kansas Medical Center. Kansas City, Kansas, USA

**Keywords:** omega-3, polyunsaturated fatty acid, dopamine receptor, postpartum, docosahexaenoic acid, rat

## Abstract

Decreased tissue levels of n-3 (*omega*-3) fatty acids, particularly docosahexaenoic acid (DHA), are implicated in the etiologies of non-puerperal and postpartum depression. This study examined the effects of a diet-induced loss of brain DHA content and concurrent reproductive status on dopaminergic parameters in adult female Long–Evans rats. An α-linolenic acid-deficient diet and breeding protocols were used to produce virgin and parous female rats with cortical phospholipid DHA levels 20–22% lower than those fed a control diet containing adequate α-linolenic acid. Decreased brain DHA produced a significant main effect of decreased density of ventral striatal D_2_-like receptors. Virgin females with decreased DHA also exhibited higher density of D_1_-like receptors in the caudate nucleus than virgin females with normal DHA. These receptor alterations are similar to those found in several rodent models of depression, and are consistent with the proposed hypodopaminergic basis for anhedonia and motivational deficits in depression.

## Introduction

Polyunsaturated fatty acids (PUFAs) are a component of the phospholipids that form the membranes of all cells. In the membrane, PUFAs serve as precursors for signaling molecules (*e.g.* prostaglandins and thromboxanes), activate transcription factors and nuclear receptors (*e.g.* PPARα and RXR), and influence the physicochemical properties of the membrane, thus affecting the function of membrane-bound proteins such as receptors and ion channels.^1^,^2^ In the brain, the n-3 (or *omega*-3) PUFA docosahexaenoic acid (DHA; 22:6n-3) is the most abundant, representing about 15% of all lipids in that tissue.^3^ DHA accumulates in the brain primarily during pre- and neonatal development; it is supplied by the mother to the developing fetus *in utero*, and to the neonate via breast milk.^4^,^5^ Inadequate accumulation of DHA in brain phospholipids does not affect overall growth or brain weight; however, sufficient DHA is necessary for optimal brain development and neuronal function.^6^,^7^

Depression occurs almost twice as commonly in females than in males,^8^ with the postpartum period representing a time of increased incidence of depression in females.^9^ A number of clinical studies of PUFAs in depression suggest a role for decreased n-3 PUFAs, particularly DHA, in the pathogenesis of the disease. Most notably, DHA content in the orbitofrontal cortex was 22% lower in postmortem patients with major depressive disorder compared to controls; this decrease was even greater in depressed females than in males.^10^ Likewise, DHA levels were lower in the adipose tissue, plasma, serum, or erythrocytes of depressed patients than controls.^11^–^16^ Epidemiological studies also indicate that lower levels of fish consumption, a major dietary source of DHA and other n-3 PUFAs, are associated with higher rates of depression.^17^–^20^ Furthermore, n-3 PUFA supplements improved depressive symptoms in several clinical trials.^21^ Similar epidemiological and clinical findings implicate decreased DHA in the etiology of postpartum depression.^22^–^26^

Decreased dopaminergic function appears to contribute to anhedonic behavior in several animal models, and has been proposed to play a role in the pathogenesis of depression (as reviewed elsewhere^27^–^30^). Of note, concentrations of the dopamine metabolite homovanillic acid (HVA) in cerebrospinal fluid were decreased in depressed patients and suicide victims, and were inversely related to depressed mood and psychomotor retardation.^31^–^34^ Depression is also common in Parkinson's disease, a neurodegenerative disorder involving the loss of nigrostriatal dopamine neurons, and can precede the onset of significant motor symptoms.^35^,^36^ Thus, decreased dopaminergic neurotransmission has been hypothesized to underlie the anhedonia and motivational deficits associated with the disorder.^37^

The percentage of DHA in brain phospholipids of adult female rats can be decreased by roughly 20%, similar to the decrease reported in depressed humans,^10^ if the animals are fed an n-3 PUFA-deficient diet for a sufficient period of time.^38^ This loss of DHA occurred more rapidly in reproducing females, presumably due to physiological demands of pregnancy and lactation.^39^ Female rats with dietinduced decreases in brain DHA exhibited decreased hippocampal brain-derived neurotrophic factor (BDNF) expression and altered regulation of the hypothalamic–pituitary–adrenal (HPA) axis similar to that reported in depression.^40^ In addition, parous dams with decreased brain DHA exhibited shorter latency to immobility in the forced swim test,^40^ an effect opposite of that produced by antidepressant drugs.^41^ While a confluence of genetic and environmental factors may be required for the development of depression,^42^ the similarity of these effects with neurobiological findings in depressed humans and in rodent models of depression suggests that a decrease in brain DHA represents one of the factors that can create a state of vulnerability that contributes to the development the disease.

In view of the evidence supporting roles for n-3 PUFAs and dopamine in the pathogenesis of depression, the higher prevalence of depression in women, and the particular vulnerability to depression conferred by the postpartum state, this study used a rat model to examine the effects of a clinically-relevant decrease in brain phospholipid DHA content and its interactions with reproductive status (virgin or parous) on the CNS dopamine systems in female rats. The effects of these treatments on gene expression of brain-derived neurotrophic factor (BDNF), a mediator of plasticity in the mesolimbic system that was increased in the nucleus accumbens of postmortem depressives^43^,^44^ and that modulates expression of dopamine receptors,^45^,^46^ was also assessed. Finally, to ascertain that the observed effects were not secondary to DHA level-induced variation in estradiol levels within the virgin or parous groups, serum estradiol concentrations were also determined. We show that a diet-induced reduction in brain DHA content results in alterations in the density of dopamine receptors independently of changes in BDNF mRNA levels or regional dopamine content in female rats and that the effects differ depending on reproductive status.

## Materials and methods

### Animals

Experiments were conducted in accordance with the NIH *Guide for the Care and Use of Laboratory Animals* and were approved by the University of Kansas Medical Center Institutional Animal Care and Use Committee.

Adult, male and female Long–Evans rats (Harlan, Indianapolis, IN, USA) were housed in a temperatureand humidity-controlled animal facility with a 12-h dark-light cycle (on at 06:00 h), and given food and water *ad libitum*. Rats were obtained at least 5 days prior to any treatments and were handled regularly. Four separate cohorts of rats were used.

### Experimental design

A between-groups design was used to assess the effects of reproductive status (virgin or parous) and brain DHA content (normal or decreased). Brain DHA content was manipulated by feeding diets varying in n-3 PUFA content to effect (see below).

Parous dams underwent two sequential reproductive cycles (gestation and nursing), with the initial mating occurring on postnatal day (P) 75–80. Dams were placed on the experimental diets at the time of the first mating. Litters were weighed and culled to eight pups on P1, and weaned on P20. The second mating occurred 8–10 days after weaning of the first litter. Parous dams were euthanized on P20 after the second litter. This treatment produces maximal alterations in brain PUFA composition in dams fed the deficient diet.^39^ Furthermore, the maternal brain was examined at the end of the period of greatest offspring demand for DHA (*i.e.* weaning), which in this respect is similar to the postpartum period in humans (1–3 months after birth)^47^,^48^ and, thus, parallels the clinical onset of postpartum depression.^49^

Virgin females with normal DHA were fed the control diet for 13 weeks beginning at P75–80. This treatment duration corresponds to the time required for two reproductive cycles and is based on our prior finding that brain PUFA composition does not vary in virgin adult females fed the control diet for periods ranging from 6 weeks to 6 months.^39^ Virgin females with decreased DHA were produced by feeding the deficient diet for 6 months, the treatment required to produce a decrease in brain DHA comparable to that observed in parous dams fed the deficient diet.^39^ Treatment of virgin females fed the deficient diet started at P56–60, thus bracketing the treatment period for the other groups. Virgin females were euthanized during diestrus, determined by vaginal lavage performed at the same time each day (09:00–10:00 h). Once regular cycling was confirmed, virgins were considered to be in diestrus on the day following estrus, and were not subjected to vaginal lavage on the day of euthanasia.

At the completion of the diet and breeding treatments, and between 10:00 and 11:00 h, rats were euthanized by decapitation. Trunk blood was collected, allowed to clot, centrifuged, and serum collected. Brains were rapidly removed and frozen on dry ice. Brain regions of interest were isolated by freehand dissection on ice according to a modification of the methods of Glowinski and Iversen.^50^ All samples were stored at –70°C.

### Experimental diets

The control diet was prepared from a purified basal mix (TD00235; Teklad, Indianapolis, IN, USA) with pure unhydrogenated soybean oil (70 g/kg), and was identical in composition to Teklad AIN-93G, which meets all current nutrient standards for rat pregnancy and growth.^51^ The deficient diet was the same as the control diet, except it was prepared with safflower oil (66.5 g/kg) and soybean oil (3.5 g/kg) as previously described.^40^ Analysis of the fatty acid composition of the diets indicated that the control diet contained 4.20 g/kg α-linoleic acid and 33.81 g/kg linoleic acid. The deficient diet contained 0.38 g/kg α-linoleic acid and 45.96 g/kg linoleic acid.

### Brain total phospholipid fatty acid composition

As previously described,^52^ lipids were extracted from occipital cortex, a brain region not required for the determination of other end-points but exhibiting dietinduced changes in DHA content similar to whole brain. Phospholipids were isolated by thin layer chromatography. The band containing total phospholipids was removed and transmethylated with boron trifluoride methanol (Sigma, St Louis, MO, USA) to yield fatty acid methyl esters. Individual fatty acid methyl esters were separated on a Varian 3400 gas chromatograph with an SP-2330 capillary column (30 m; Supelco, Inc., Belfonte, PA, USA), with helium used as the carrier gas. The resulting peaks were identified by comparison to authentic standards (PUFA 1 and PUFA 2, Supelco, Inc.; 22:5n-6, Nu-Chek Prep, Elysian, MN, USA) and expressed as weight percent of total fatty acids on the basis of peak areas of individual fatty acids in a mixture of fatty acids of known weight (Supelco 37 mixture).

### Receptor binding assays

Dissected brain tissue was homogenized in 20 vol (w/v) of buffer (50 mM Tris; pH 7.4 at 23°C) using a PRO homogenizer (setting 4 for 10 s). The crude homogenate was centrifuged twice (15 min at 48,000 *g*), and pellet resuspended each time in 20 vol of buffer. The final pellet was resuspended in the appropriate concentration for each assay in the respective assay buffer.

The affinity and density of D_1_-like receptors were assessed by Scatchard analysis using four concentrations of [*N*-methyl-^3^H]-SCH 23390 (87 Ci/mmol; 0.04–1.2 nM; GE Healthcare, Piscataway, NJ, USA) in an equilibrium, filtration assay as previously described.^53^ The assay buffer was 50 mM Tris, 120 mM NaCl, 5 mM KC1, 2 mM CaCl_2_, 1 mM MgCl_2_, pH 7.4 at 23°C. Non-specific binding was defined in the presence of 1 μM (+)-butaclamol. Membrane protein concentrations were determined by the BCA method (Pierce, Rockford, IL, USA) and were ∼50 μg/tube. Assay tubes were incubated at 23°C for 90 min. The reaction was terminated by rapid vacuum filtration. Bound radioactivity was quantified using a Beckman LS-6500 liquid scintillation counter. Specific binding is expressed as fmol/mg protein and analyzed for K_D_ and B_max_ using SigmaPlot (v8.0.2) in accordance with the methods of Bylund and Yamamura.^54^

The affinity and density of D_2_-like receptors were assessed using [^3^H]-spiperone (21 Ci/mmol; 0.03–0.7 nM; GE Healthcare) as previously described.^53^ Assays were performed as described for D_1_-like receptor binding except the assay buffer was 50 mM Tris, 5 mM KC1, 2 mM CaCl_2_, 1 mM MgCl_2_, pH 7.4 at 23°C.

D_3_-receptor binding was assessed using [^3^H]-PD 128907 as previously described.^55^ Due to the limited quantity of ventral striatal tissue and the low density of D_3_ sites, the initial binding analysis was performed using a single concentration of (+)-[*N*-propyl-2,3-^3^H]-PD 128907 (112 Ci/mmol; 0.7 nM; Amersham, Arlington Heights, IL, USA). Assays were performed as described above except the assay buffer was 50 mM Tris, 1 mM EDTA, pH 7.4 at 23°C; non-specific binding was defined by 1 μM spiperone, the membrane protein concentration was ∼200 μg/tube, and the incubation time was 3 h. Because no significant differences were observed between groups in the single-point analysis, further assessment of D_3_ binding by Scatchard analysis was not performed.

### Measurement of monoamine neurotransmitters and metabolites

Concentrations of dopamine, dihydroxyphenylacetic acid (DOPAC), and HVA were quantified using an isocratic HPLC-EC system (ESA Coulochem III, Chelmsford, MA, USA) coupled to a Coulochem III dual-channel electrochemical array detector (ESA Inc., Chelmsford, MA; Model 5100A, E_1_–150 mV and E_2_ + 275 mV using a 5011 dual analytical cell) as previously described.^40^ Tissues were extracted in perchloric acid, diluted with mobile phase, and analytes separated using a C18 reverse phase column (ESA Inc., HR-80, 4.6 mm × 80 mm, 3 μm particles) with a citrate-acetate mobile phase containing 6.0% methanol and 0.35 mM l-octane-sulfonic acid (pH 4.1). The flow rate was 1.8 ml/min. The internal standard was 3,4-dihydroxybenzylamine. Protein concentration of the extracted tissue was determined by the BCA method (Pierce). Monoamine concentrations are expressed as nanogram per milligram protein. Neurotransmitter turnover was calculated as the ratio of the metabolites to the neurotransmitter.

### Solution hybridization – nuclease protection assays for BDNF mRNAs

Total cellular RNAs were extracted from ventral striatal and caudate nucleus tissue samples using a rapid guanidinium isothiocyanate phenol/chloroform method. The resultant total RNAs were analyzed for BDNF and β-actin mRNA levels by solution hybridization – nuclease protection assays using [^32^P]-labeled antisense cRNA probes as previously described.^56^,^57^ Specific mRNAs were quantified by comparison to cRNA quantitation standards and are reported as picogram BDNF mRNA per nanogram β-actin mRNA.

### Estradiol radioimmunoassay

Serum estradiol concentrations were determined using an estradiol Coat-a-Count radioimmunoassay kit (Siemens/Diagnostic Products Corp., Los Angeles, CA, USA).

### Data analysis

Reproductive status and brain phospholipid DHA content were used as the descriptors for the independent variables, reflecting the experimental approach of subjecting the rats to the diet treatment to effect, rather than for a specified period of time. Data are expressed as the mean ± SEM. Data were analyzed for statistically significant effects by two-way ANOVA with factors of brain DHA content and reproductive status (Systat, v10.2). Significant effects were further analyzed *post-hoc* using one-way ANOVA with all treatment groups, followed by the Tukey–Kramer multiple comparisons test. Differences between groups were considered significant at *P* < 0.05.

## Results

### Cortical phospholipid fatty acid composition

Treatment of virgin females with the deficient diet for 6 months decreased the percentage of DHA in cortical phospholipids by 20% compared to virgin females fed the control diet. The decrease in DHA was accompanied by a 6.4-fold compensatory increase in the percentage of docosapentaenoic acid (DPA; 22:5n-6) compared to virgin females fed the control diet. The percentage of arachidonic acid (20:4n-6) was not altered. These diet-induced changes in brain fatty acid composition concur with previous studies.^39^,^40^,^58^ Similar changes in phospholipid fatty acid composition were observed in parous dams fed the diets to the period of time required to complete two reproductive cycles, also in agreement with previous studies.^39^,^40^ Representative data from one of the four cohorts of rats used in these experiments are shown in Table 1.

**Table 1:** Effects of diet and breeding treatments on the percentage of docosahexaenoic acid (DHA; 22:6n-3), docosapentaenoic acid (DPA; 22:5n-6), and arachidonic acid (AA; 20:4n-6) in cortical phospholipids of female rats

Group	DHA	DPA (Percentage of total fatty acids)	AA
Virgin–normal DHA	15.0 ± 0.32	0.31 ± 0.014	10.8 ± 0.16
Virgin–decreased DHA	12.1 ± 0.35^a^	2.3 ± 0.027^a^	11.3 ± 0.15
Parous–normal DHA	14.6 ± 0.71^b^	0.46 ± 0.24^b^	11.8 ± 0.38
Parous–decreased DHA	11.5 ± 0.33^a^,^b^	2.2 ± 0.090^a^,^c^	11.6 ± 0.42

Data are presented as the mean ± SEM (*n* = 6 or 7 per group) and are representative of those obtained from one of the four cohorts of rats used in these studies.

a Different from Virgin–normal DHA (*P* < 0.05).

b Different from Virgin-decreased DHA (*P* < 0.05).

c Different from Parous–normal DHA (*P* < 0.05).

Virgin females with normal DHA were fed the control diet for 13 weeks, corresponding to two reproductive cycles, beginning at P75–80. Virgin females with decreased DHA were produced by feeding the deficient diet for 6 months, starting at P56–60, thus bracketing the treatment period for the other groups. Parous dams underwent two sequential reproductive cycles (gestation and nursing), beginning at P75–80, while being fed either the control or deficient diets.

### Body weight

**Figure 1:**
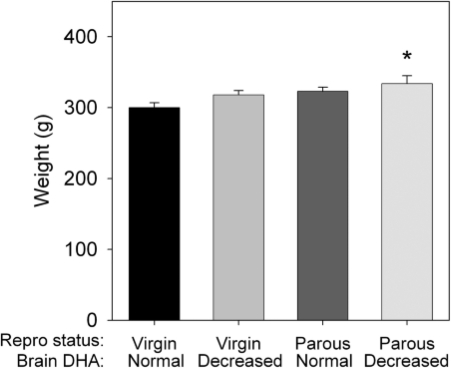
Effects of diet and breeding treatments on body weights in female rats. Data are presented as the mean ± SEM (*n* = 15 per group). Body weight was determined at the completion of the diet and breeding treatment immediately prior to euthanasia, and is representative of those obtained from each of the four cohorts of rats used in these studies. *Different from Virgin–normal DHA (*P* < 0.05). These data were previously reported in Levant *et al.*^40^

As previously reported,^40^ a significant main effect of reproductive status on body weight at the end of the diet and breeding treatments was indicated by two-way ANOVA (*P* < 0.05), with parous dams having 6% higher body weight than virgin females. There was no main effect of diet treatment (Fig. 1).

### Regional dopamine receptor binding

In the ventral striatum (nucleus accumbens and olfactory tubercle), two-way ANOVA indicated a significant main effect of brain DHA content on D_2_-like receptor binding density (B_max_; *P* < 0.01), and significant main effects of reproductive status (*P* < 0.05) and brain DHA content (*P* < 0.01) on D_2_-like receptor affinity (K_D_; Table 2). *Post-hoc* analysis indicated that the density of D_2_-like receptors in ventral striatum was 37% lower in parous dams with decreased DHA than in parous dams with normal DHA (*P* < 0.05). The density of D_2_-like receptors in the ventral striatum of virgin females with decreased DHA was 27% lower than in virgin females with normal DHA, but was not quite statistically significant (*P* = 0.069). The affinity of D_2_-like sites in this brain region was roughly 2-fold lower in parous dams with normal DHA than in parous dams with decreased DHA (*P* < 0.05) or in virgin females with decreased DHA (*P* < 0.05). The affinity and density of D_1_-like receptors in the ventral striatum were not affected by reproductive status or brain DHA content (Table 3). Single-point binding assays with [^3^H]-PD 128907 indicated that the concentrations of D_3_ receptors in the ventral striatum were 16.1 ± 0.54 fmol/mg protein in the virgin-normal DHA group, 16.2 ± 1.23 fmol/mg protein in the virgin-decreased DHA group, 16.4 ± 0.74 fmol/mg protein in the parous-normal DHA group, and 15.5 ± 1.16 fmol/mg protein in the parous-decreased DHA group; and were not different between groups.

**Table 2:** Effects of brain DHA content and reproductive status on the affinity and density of dopamine receptors in the ventral striatum (nucleus accumbens and olfactory tubercle) and caudateputamen

Reproductive status/brain DHA content	B_max_(fmol/mg protein)	K_D_(nM)
Ventral striatum		
D_1_-like receptors		
Virgin–normal DHA	455 ± 33	0.27 ± 0.010
Virgin–decreased DHA	470 ± 25	0.25 ± 0.012
Parous–normal DHA	483 ± 7	0.23 ± 0.012
Parous–decreased DHA	429 ± 21	0.24 ± 0.019
D_2_-like receptors		
Virgin–normal DHA	194 ± 18	0.29 ± 0.047
Virgin–decreased DHA	141 ± 18	0.23 ± 0.036
Parous–normal DHA	239 ± 36^b^	0.50 ± 0.072^b^
Parous–decreased DHA	150 ± 20^c^	0.26 ± 0.057^c^
Caudate nucleus		
D_1_-like receptors		
Virgin–normal DHA	501 ± 27	0.19 ± 0.010
Virgin–decreased DHA	599 ± 24^a^	0.22 ± 0.008
Parous–normal DHA	615 ± 20^a^	0.21 ± 0.009
Parous–decreased DHA	567 ± 14	0.18 ± 0.007^b^
D_2_-like receptors		
Virgin–normal DHA	276 ± 23	0.22 ± 0.030
Virgin–decreased DHA	251 ± 11	0.23 ± 0.024
Parous–normal DHA	268 ± 25	0.20 ± 0.031
Parous–decreased DHA	289 ± 21	0.22 ± 0.027

Data are presented as the mean ± SEM (*n* = 7–10 per group). D_1_-like receptor binding was determined using [^3^H]-SCH 23390. D_2_-like receptor binding was determined using [^3^H]-spiperone.

a Different from Virgin–normal DHA (*P* < 0.05).

b Different from Virgin-decreased DHA (*P* < 0.05).

c Different from Parous–normal DHA (*P* < 0.01).

**Table 3:** Effects of brain DHA content and reproductive status on the concentrations of dopamine, DOPAC, and HVA in ventral striatum (nucleus accumbens and olfactory tubercle), caudate-putamen, and substantia nigra/ventral tegmental area

Reproductive status/brain DHA content	Dopamine	DOPAC	HVA	Turnover
	(ng/mg protein)			
Ventral striatum				
Virgin–normal DHA	40 ± 3.2	15 ± 0.70	25 ± 2.2	1.1 ± 0.10
Virgin–decreased DHA	39 ± 3.1	18 ± 1.3	26 ± 1.4	1.2 ± 0.09
Parous–normal DHA	44 ± 3.8	17 ± 1.0	25 ± 1.6	1.1 ± 0.12
Parous–decreased DHA	42 ± 2.5	15 ± 1.0	24 ± 1.6	1.0 ± 0.11
Caudate nucleus				
Virgin–normal DHA	84 ± 5.0	21 ± 1.9	26 ± 0.69	0.59 ± 0.06
Virgin–decreased DHA	88 ± 8.6	23 ± 2.1	27 ± 1.6	0.61 ± 0.06
Parous–normal DHA	84 ± 5.9	22 ± 2.8	28 ± 2.6	0.59 ± 0.04
Parous–decreased DHA	100 ± 8.9	22 ± 1.6	27 ± 1.8	0.52 ± 0.04
Substantia nigra/ventral tegmental area				
Virgin–normal DHA	9.0 ± 0.70	6.5 ± 1.0	12 ± 0.72	2.3 ± 0.36
Virgin–decreased DHA	7.6 ± 0.71	7.5 ± 0.90	11 ± 0.84	2.8 ± 0.46
Parous–normal DHA	9.7 ± 0.99	6.5 ± 0.73	11 ± 0.95	2.2 ± 0.43
Parous–decreased DHA	9.5 ± 1.1	5.9 ± 0.77	11 ± 0.66	2.2 ± 0.49

Data are presented as the mean ± SEM (*n* = 10 or 11 per group). No significant differences between groups were detected by ANOVA.

In the caudate-putamen, two-way ANOVA indicated a significant interaction of reproductive status and brain DHA content on D_1_-like receptor density (*P* < 0.01) and affinity (*P* < 0.01; Table 3). *Post-hoc* analysis indicated that the density of D_1_-like receptors in the caudate-putamen was 20% higher in virgin females with decreased DHA compared to virgin females with normal DHA (*P* < 0.05). The density of D_1_-like receptors was also 23% higher in parous dams with normal DHA compared to virgin females with normal DHA (*P* < 0.05). In addition, the affinity of D_1_-like receptors was 10% higher in parous dams with decreased DHA compared to virgin females with decreased DHA (*P* < 0.05). The affinity and density of D_2_-like receptors in the caudateputamen were not affected by reproductive status or brain DHA content.

### Regional dopamine concentrations and turnover

No effects of reproductive status or brain DHA content were detected on the concentrations of dopamine and its metabolites in the ventral striatum, caudate-putamen, or substantia nigra/ventral tegmental area, nor were there any significant differences in dopamine turnover (Table 3).

### BDNF gene expression

No effects of reproductive status or brain DHA content were detected on the concentrations of BDNF mRNA in the ventral striatum or caudate-putamen (Table 4).

**Table 4:** Effects of brain DHA content and reproductive status on the concentrations of BDNF mRNA in ventral striatum (nucleus accumbens and olfactory tubercle) and caudate-putamen

Reproductive status/brain DHA content	BDNF mRNA(pg/ng β-actin mRNA)
Ventral striatum	
Virgin–normal DHA	11.5 ± 2.1
Virgin–decreased DHA	12.1 ± 3.2
Parous–normal DHA	12.5 ± 1.7
Parous–decreased DHA	14.6 ± 1.3
Caudate nucleus	
Virgin–normal DHA	16.6 ± 2.2
Virgin–decreased DHA	16.0 ± 2.0
Parous–normal DHA	14.2 ± 1.6
Parous–decreased DHA	16.0 ± 1.1

Data are presented as the mean ± SEM (*n* = 10–12 per group). No significant differences between groups were detected by ANOVA.

### Serum estradiol

**Figure 2:**
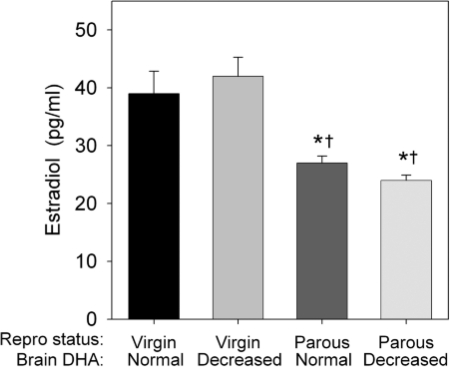
Effects of brain DHA content and reproductive status on serum estradiol concentrations. Data are presented as the mean ± SEM (*n* = 9–12 per group). *Different from Virgin–normal DHA (*P* < 0.05). †Different from Virgin–decreased DHA (*P* < 0.05)

Two-way ANOVA indicated a significant main effect of reproductive status on serum estradiol concentrations (*P* < 0.001), which were 30–45% lower in parous dams than in virgin females. There was no effect of brain DHA content on estradiol levels between the virgin or parous groups (Fig. 2).

## Discussion

The effects of a diet-induced reduction of brain phospholipid DHA content was assessed in diestrus virgin female rats and in bi-parous dams at the time of weaning. The present data demonstrate alterations in regional dopamine receptor density, thus extending findings on the neurobiological effects of a dietinduced decrease in brain DHA content in adult female animals to the dopamine system. Levels of BDNF mRNA in the ventral striatum (nucleus accumbens and olfactory tubercle) and caudateputamen were not altered by the diet and breeding treatments, indicating that modulation of gene expression for this neurotrophic factor differs in these brain regions from hippocampus, where these treatments increased expression of BDNF.^40^ Although protein concentrations need not necessarily reflect levels of mRNA, the present data suggest that the effects of the diet-induced changes in brain DHA produced in this study differ from findings in depression where BDNF concentrations were decreased in the nucleus accumbens.^43^ Serum estradiol levels were not different between diet groups. Accordingly, neither the dopamine receptor alterations reported here, nor the previously reported effects such as altered hippocampal BDNF expression and HPA axis regulation,^40^ can be attributed to dietinduced variation in estradiol levels within the virgin or parous groups or to early re-instatement of estrous cycling in the lactating dams. Furthermore, previous findings rule out diet-induced variation in overall health, weight gain, and maternal offspring burden as contributing factors in the observed effects.^40^

A diet-induced decrease brain DHA content produced a decrease in the density of D_2_-like dopamine receptors in the ventral striatum, which was greater in magnitude in parous dams than in virgin females, where the effect was not quite significant. This observation is consistent with the proposed hypo-activity of the mesolimbic dopamine system in depression.^27^,^29^,^30^ However, a recent study in drug-naïve patients with major depressive disorder found no differences in the density of D_2_ receptors in either the ventral striatum or the caudate nucleus.^59^ Even so, densities of D_2_-like receptors or mRNA in the nucleus accumbens were decreased in several putative rodent models of depression such as chronic mild stress-induced anhedonia and the socially-isolated Flinders sensitive line rat,^60^,^61^ suggesting that hypo-activity of the mesolimbic dopamine system may have differing underlying bases in humans and rodents. The Wistar–Kyoto rat, another rodent depression model, also exhibited decreased D_2_ binding in the nucleus accumbens core compared to the Wistar progenitor strain, though D_2_ binding was also increased in the nucleus accumbens shell.^62^ In addition, decreased density of D_2_ receptors in the nucleus accumbens was detected in rats subjected to inescapable stress in the learned helplessness model, but was present regardless of whether the rats exhibited subsequent learned helplessness behaviors.^63^ Some of these putative animal models of depression also exhibited increased^61^,^63^ or decreased^64^ levels of D_1_ receptor binding or mRNA in this brain region, which was not altered in either virgin or parous rats with decreased brain DHA.

In addition to the changes in ventral striatal D_2_-like receptor density, virgin females with a diet-induced decrease in brain DHA content exhibited increased density of D_1_–like receptors in the caudate-putamen. An increase in D_1_–like receptors in this brain region, without an alteration in D_2_ receptor density was also reported in the chronic mild stress-induced anhedonia model of depression.^60^ Likewise, higher levels of D_1_ receptor mRNA were detected in the caudateputamen of the Flinders sensitive line rat, with or without social isolation, although this rodent depression model also exhibited decreased density of D_2_ receptor mRNA in this brain region when socially isolated.^61^ In the inescapable stress-learned helplessness model, an increase in D_1_ binding in the caudate-putamen, with no change in D_2_ binding, was observed after inescapable stress, but only in the subset of rats that did not exhibit subsequent learned helplessness.^63^ However, D_1_ and D_2_ binding in the caudate-putamen were both lower in the Wistar–Kyoto rat depression model,^62^,^64^ and lower densities of D_1_ receptors were observed in the caudate nucleus of patients with major depression in PET imaging studies,^65^,^66^ suggesting that further research is required to resolve the contribution of specific dopamine receptor alterations in this brain region to the depressive phenotype.

Dopamine receptor affinity and density are known to be regulated by dopaminergic neurotransmission and by BDNF.^45^,^46^,^67^ However, the changes in dopamine receptor density observed in this study occurred in the absence of concurrent changes of regional dopamine concentration and turnover or changes in BNDF gene expression, indicating that other mechanism are involved. A decrease in brain DHA content, which occurs throughout the brain, affects a variety of systems by multiple mechanisms involving altered membrane properties, changes in intra- and intercellular signaling, and modulation of gene transcription (see Introduction).^1^,^2^ The receptor changes reported here thus likely represent the net effect of these changes, the specific nature of which must be determined in future studies.

## Conclusions

The present findings demonstrate that a diet-induced reduction in brain DHA content in female rats results in alterations in the density of dopamine receptors that are similar to those found in several rodent models of depression, differ depending on reproductive status, and occur independently of changes in BDNF mRNA levels or regional dopamine content.
